# Supplementary material for: What does it take to learn the rules of RNA base pairing? A lot less than you may think
Source: bioRxiv. 2025 Aug 2:2025.07.31.668042. Preprint. [Version 1] doi: 10.1101/2025.07.31.668042 (PMC12324431; doi:10.1101/2025.07.31.668042)
Supplement: Supplement 1 [file media-1.gz › supplemental_material/experiments/g6_optimize_param_conus_rnabench_RNaseP_random/g6_params_i88.pdf]

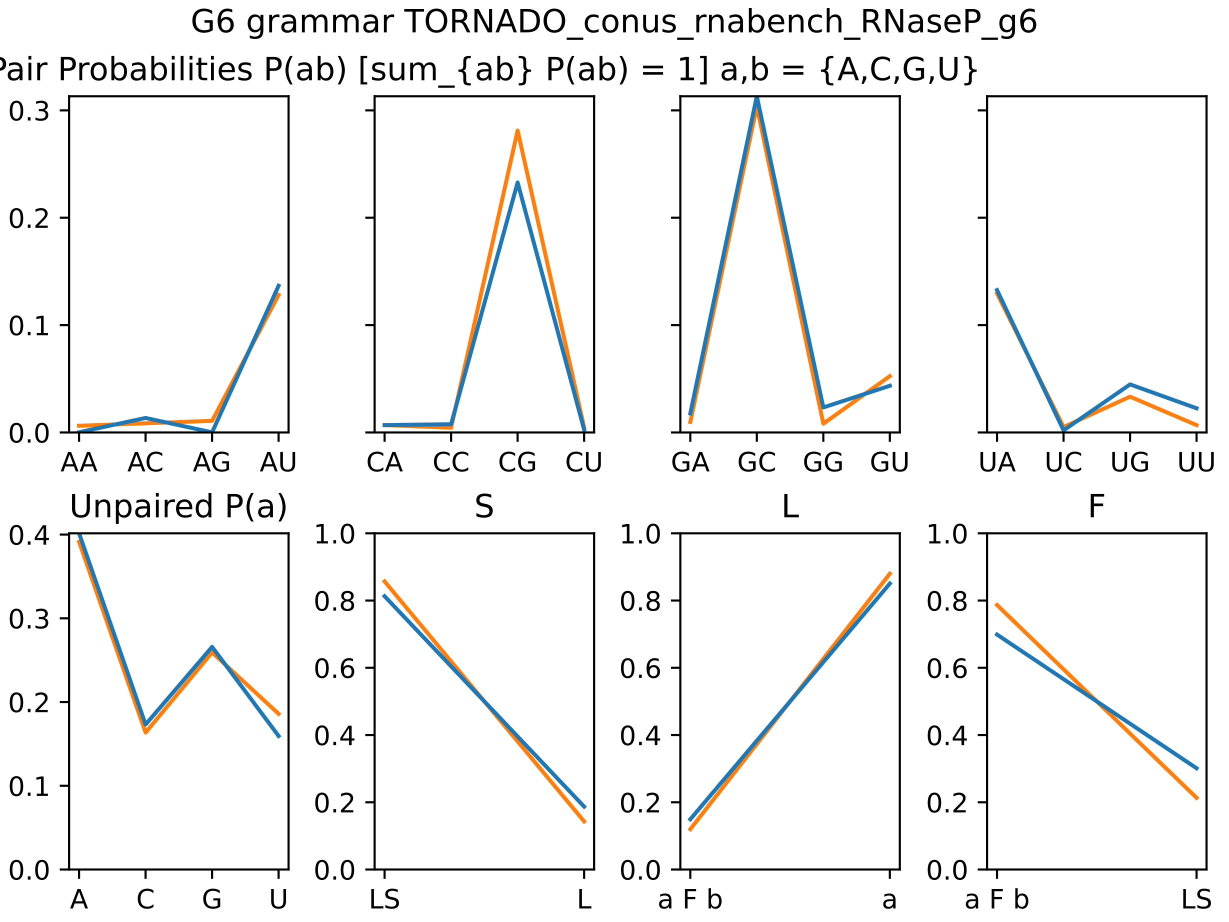
G6 grammar TORNADO_conus_rnabench_RNaseP_g6
Pair Probabilities P(ab) [sum_{ab} P(ab) = 1] a,b = {A,C,G,U}
0.0
0.1
0.2
0.3
AA
AC
AG
AU
CA
CC
CG
CU
GA
GC
GG
GU
UA
UC
UG
UU
Unpaired P(a)
0.0
0.1
0.2
0.3
0.4
A
C
G
U
S
LS
L
L
a F b
a
F
a F b
LS
0.0
0.2
0.4
0.6
0.8
1.0
